# The impact of severe mental illness (SMI) on the rate of COVID-19 vaccine uptake and hesitancy

**DOI:** 10.1192/j.eurpsy.2023.977

**Published:** 2023-07-19

**Authors:** T. Hamakarim, A. Arumuham, S. Parmar, O. D. Howes

**Affiliations:** 1 GKT School of Medical Education, King’s College London, London, UK; 2 Department of Psychosis Studies, Institute of Psychiatry, Psychology & Neuroscience, King’s College London; 3 Institute of Clinical Sciences (ICS), Faculty of Medicine, Imperial College London; 4 South London and Maudsley NHS Foundation Trust, London; 5 H Lundbeck A/s, St Albans, United Kingdom

## Abstract

**Introduction:**

The COVID-19 pandemic has disproportionately affected patients with severe mental illness (SMI), a vulnerable population with high morbidity and mortality. A UK-based study found reduced vaccination rates in patients with SMI; they therefore need to be prioritised for prevention and disease management.

**Objectives:**

The objectives were to determine risk factors for vaccine hesitancy, and how best to manage those in patients with SMI, as well as whether our intervention of calling patients for their vaccines had a positive outcome.

**Methods:**

Following approval from the Lambeth Directorate of South London and Maudsley (SLaM) NHS Foundation Trust, we investigated COVID-19 vaccination rates inpatients with SMI from a psychosis community service in South London (n=236). Dates of first and second doses were recorded through audit; reasons for refusal of vaccination were noted. Patients were encouraged to take the vaccine. A re-audit was performed after allowing three months. Chi-squared statistical analysis was performed to determine the value of our intervention.

**Results:**

Before the intervention, 143 patients (60.6%) received at least one vaccine. 24 patients (10.2%) received one dose, and 77 (32.8%) were yet to receive any. There was no statistical significance (p=0.1509) between the number of patients who received a vaccine before and after intervention, with 33.1% of patients still remaining unvaccinated.

**Image:**

**Figure:**
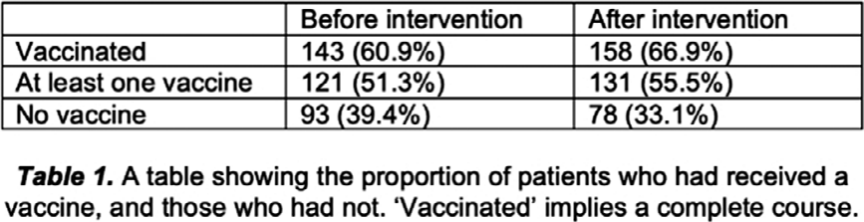

**Image 2:**

**Figure:**
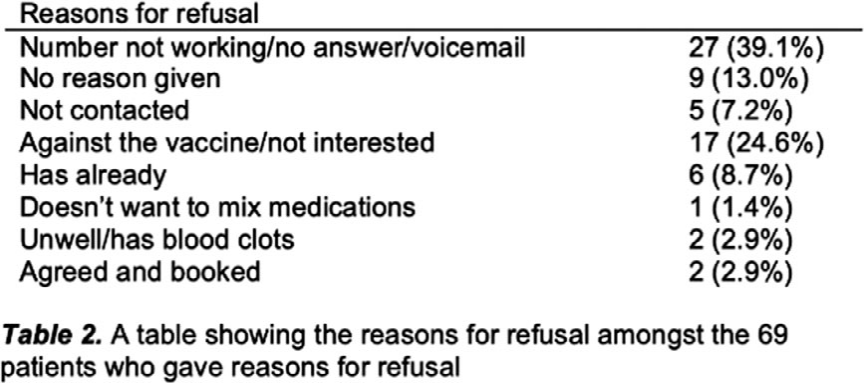

**Image 3:**

**Figure:**
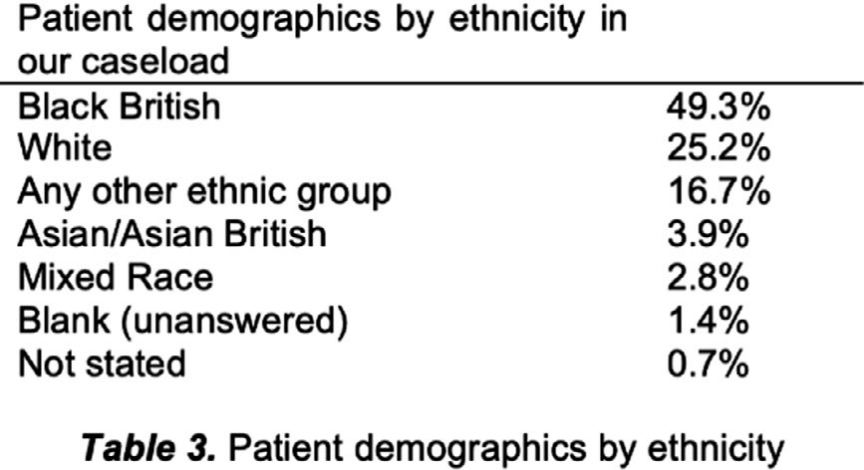

**Conclusions:**

There is limited research on perceptions of receiving vaccines in patients with SMI, despite their cost-effectiveness in disease prevention. Even after intervention, 33.1% of patients remained unvaccinated, compared to 6.6% nationally. A lack of knowledge and recommendations from care teams are reasons for hesitancy. Misinformation, conspiracy theories and propaganda can drive people towards refusal. Patients with SMI typically have disadvantages of healthcare inequality, lower levels of education and access to inaccurate information. Patients and their healthcare team should be knowledgeable about vaccine efficacy and side effects. Studies have shown low uptake in the Black/African/Caribbean ethnic group (49.3%, table 3). Reasons include general mistrust in institutions and access barriers. For minority communities, vaccination sites in community centres or places of worship have proven to be effective, providing familiarity.

Patients taking clozapine may have a weaker immune system due to myelosuppression. 24.3% of our patients take this, with many unsure of interactions or side effects. Poorer prognosis means a focussed approach is needed.

Vaccine hesitancy is complex and requires targeted, tailor-made strategies, with consideration for patients who may lack capacity. It is evident from our results that calling patients alone may not be effective. A future multi-modal approach may be necessary to address poor vaccine uptake and opens up avenues to further explore vaccine hesitancy.

**Disclosure of Interest:**

None Declared

